# Adult and adolescent exposure to tobacco and alcohol content in contemporary YouTube music videos in Great Britain: a population estimate

**DOI:** 10.1136/jech-2015-206402

**Published:** 2016-01-14

**Authors:** Jo Cranwell, Magdalena Opazo-Breton, John Britton

**Affiliations:** Division of Epidemiology and Public Health, UK Centre for Tobacco and Alcohol Studies, University of Nottingham, Nottingham, UK

**Keywords:** ALCOHOL, Tobacco, PUBLIC HEALTH

## Abstract

**Background:**

We estimate exposure of British adults and adolescents to tobacco and alcohol content from a sample of popular YouTube music videos.

**Methods:**

British viewing figures were generated from 2 representative online national surveys of adult and adolescent viewing of the 32 most popular videos containing content. 2068 adolescents aged 11–18 years (1010 boys, 1058 girls), and 2232 adults aged 19+years (1052 male, 1180 female) completed the surveys. We used the number of 10 s intervals in the 32 most popular videos containing content to estimate the number of impressions. We extrapolated gross and per capita impressions for the British population from census data and estimated numbers of adults and adolescents who had ever watched the sampled videos.

**Results:**

From video release to the point of survey, the videos delivered an estimated 1006 million gross impressions of alcohol (95% CI 748 to 1264 million), and 203 million of tobacco (95% CI 151 to 255 million), to the British population. Per capita exposure was around 5 times higher for alcohol than for tobacco, and nearly 4 times higher in adolescents, who were exposed to an average of 52.1 (95% CI 43.4 to 60.9) and 10.5 (95% CI 8.8 to 12.3) alcohol and tobacco impressions, respectively, than in adults (14.1 (95% CI 10.2 to 18.1) and 2.9 (95% CI 2.1 to 3.6)). Exposure rates were higher in girls than in boys.

**Conclusions:**

*YouTube* music videos deliver millions of gross impressions of alcohol and tobacco content. Adolescents are exposed much more than adults. Music videos are a major global medium of exposure to such content.

## Introduction

It is well established that young people exposed to depictions of tobacco and alcohol content in films are more likely to start smoking or to consume alcohol,^1–8^ but the effect of imagery in other media, including new online media such as *YouTube* music videos, has received relatively little attention. It is becoming clear, however, that some music videos contain substantial tobacco and alcohol imagery,^9–11^ including branding, and that these portrayals are often positive.^10^

In the UK, paid-for placement of tobacco products in music videos is prohibited by the Tobacco Advertising and Promotion Act 2002 (TAPA), but this law does not apply to videos produced outside the UK.^12^ Alcohol promotion is much less tightly controlled, being subject only to a code of conduct issued by the independent Advertising Standards Authority (ASA) and by voluntary codes or practice, including that produced by the Portman Group, a not-for-profit organisation funded by 11 member companies accounting for more than half the UK alcohol market.^13^ ^14^ All are broadly consistent with ASA guidance that “Marketing communications for alcoholic drinks should not be targeted at people under 18 and should not imply, condone or encourage immoderate, irresponsible or anti-social drinking”.^15^

Since music videos tend to be most popular with younger audiences, it is most likely that tobacco and alcohol content promote use of both of these products, and that paid-for alcohol content contravenes these codes of practice. However, the extent to which adults and adolescents are exposed to tobacco or alcohol content from *YouTube* or other online music has not been quantified. We have therefore estimated population exposure to tobacco and alcohol impressions, defined as appearances in 10 s intervals in a sample of popular videos, in the British adolescent and adult population.

## Methods

### Procedure

#### Video content data

Video content data were taken from semiquantitative interval coding of tobacco and alcohol content in a previously reported sample of 110 *YouTube* music videos from the most popular top 40 chart songs in the UK in the 12 weeks from Sunday 3 November 2013 to Sunday 19 January 2014.^9^ Alcohol or tobacco actual use, implied use, paraphernalia and brand appearances were each coded as present or absent in visual imagery and lyrical content in successive 10 s intervals of the video. The interval coding system was a slightly modified version of that previously successfully used in television and film, the details of which we discuss elsewhere.^9^ ^16^ ^17^ For the present study, we combined these exposure categories into a single measure of any alcohol or tobacco content in each interval, and selected the 32 most popular videos by chart position containing either tobacco or alcohol imagery for inclusion in our surveys. These 32 videos contained a total of 821 10 s intervals for analysis, of which 47 and 233, respectively, included tobacco and alcohol content. Electronic cigarettes appeared in six intervals. Our financial resources allowed for the inclusion of 32 of the 40 videos only.

#### Survey data

We measured exposure to these 32 videos in separate online surveys of adolescents and adults carried out by YouGov PLC. The survey of adolescents, which was carried out in conjunction with the public health charity Action on Smoking and Health (ASH), involved 2068 young people (1010 boys and 1058 girls) aged 11–18 years surveyed between 21 March and 1 April 2014, and headline results have been reported in our previous study.^9^ The adult survey involved 2232 adults (1052 males and 1180 females) aged 19 years and over and was carried out on 14 and 15 July 2014. The surveys were designed to reduce coverage bias and to be representative by age, gender and region for adolescents and by age, gender, region, social grade and newspaper readership for adults. All participants were asked if they had ever seen each of the 32 videos selected for the survey. Both YouGov surveys were carried out in accordance with the Market Research Society (MRS) and the British Polling Council (BPC).

### Statistical analyses

We combined the survey data to estimate proportions of the UK population, broken down by age and gender, that had seen the videos; the video content data with the number of intervals containing any alcohol or tobacco content by video; and official UK population mid-year estimates (2012–2013) by age and gender to compute gross impressions for the whole British population.^18^ ^19^ Per capita gross impressions were obtained by dividing total gross impressions by the population in each age and gender group. All analyses were carried out using Stata V.13.^20^

## Results

Our results suggest that, at the point of survey, the proportion of adolescents who had viewed the 32 music videos was 0.22 (95% CI 0.19 to 0.26), and of adults 0.06 (95% CI 0.04 to 0.08, table 1), and that the videos delivered a total of 1006 million alcohol gross impressions (95% CI (748 to 1264 million), and 203 million tobacco gross impressions (95% CI 151 to 255 million), to the British population. On average, the videos were available for 7.1 and 10.6 months for adolescents and adults, respectively, since the date of the video release. The majority of gross impressions were delivered to adults, and particularly those in the 25–34 age group. However, the proportions of the population exposed were highest in adolescents, and per capita impressions were nearly four times higher, at 52.11 (95% CI 43.36 to 60.86) and 10.51 (95% CI 8.75 to 12.28) for alcohol and tobacco, respectively, in adolescents than in adults (14.13 (95% CI 10.21 to 18.05) and 2.85 (95% CI 2.06 to 3.64), respectively, table 1).^i^ The general characteristics of the percentage of videos watched are presented in online supplementary appendix 1.

**Table 1:** Gross impressions in millions and per capita on music video watching (at least once) for the UK population by gender and age group

			Gross impressions (in millions)		Gross impressions (per capita)	
	Population (in millions)	Proportion viewed (95% CI)	Alcohol (95% CI)	Tobacco (95% CI)	Alcohol (95% CI)	Tobacco (95% CI)
Adolescents	5.91	0.22 (0.19 to 0.26)	308.19 (256.44 to 359.93)	62.17 (51.73 to 72.60)	52.11 (43.36 to 60.86)	10.51 (8.75 to 12.28)
Female	2.88	0.28 (0.23 to 0.33)	186.35 (152.98 to 219.71)	37.59 (30.86 to 44.32)	64.66 (53.09 to 76.24)	13.04 (10.71 to 15.38)
Male	3.03	0.17 (0.14 to 0.20)	118.14 (98.05 to 138.23)	23.83 (19.78 to 27.88)	38.96 (32.33 to 45.58)	7.86 (6.52 to 9.19)
Adults	49.20	0.06 (0.04 to 0.08)	695.09 (502.38 to 887.80	140.21 (101.34 to 179.08)	14.13 (10.21 to 18.05)	2.85 (2.06 to 3.64)
Female	25.18	0.07 (0.05 to 0.09)	429.15 (311.65 to 546.66)	86.57 (62.86 to 110.27)	17.04 (12.38 to 21.71)	3.44 (2.50 to 4.38)
Male	24.02	0.05 (0.03 to 0.06)	260.81 (184.23 to 337.39)	52.61 (37.16 to 68.06)	10.86 (7.67 to 14.05)	2.19 (1.55 to 2.83)
Age groups						
11–12	1.38	0.19 (0.15 to 0.23)	61.96 (49.18 to 74.75)	12.50 (9.92 to 15.08)	44.88 (35.62 to 54.14)	9.05 (7.18 to 10.92)
13–15	2.21	0.24 (0.21 to 0.28)	125.87 (107.75 to 144.00)	25.39 (21.73 to 29.05)	56.93 (48.73 to 65.13)	11.48 (9.83 to 13.14)
16–18	2.32	0.22 (0.18 to 0.27)	120.94 (97.21 to 144.67)	24.40 (19.61 to 29.18)	52.07 (41.85 to 62.29)	10.50 (8.44 to 12.56)
19–24	5.12	0.15 (0.11 to 0.19)	178.34 (133.57 to 223.10)	35.97 (26.94 to 45.00)	34.80 (26.07 to 43.54)	7.02 (5.26 to 8.78)
25–34	8.68	0.10 (0.07 to 0.13)	205.92 (150.04 to 261.80)	41.54 (30.27 to 52.81)	23.73 (17.29 to 30.17)	4.79 (3.49 to 6.09)
35–44	8.46	0.07 (0.05 to 0.09)	138.48 (93.95 to 183.02)	27.93 (18.95 to 36.92)	16.36 (11.10 to 21.63)	3.30 (2.24 to 4.36)
45–54	9.03	0.04 (0.03 to 0.06)	93.62 (63.13 to 124.11)	18.88 (12.73 to 25.03)	10.37 (6.99 to 13.74)	2.09 (1.41 to 2.77)
55+	17.90	0.02 (0.01 to 0.03)	80.72 (53.14 to 108.31)	16.28 (10.72 to 21.85)	4.51 (2.97 to 6.05)	0.91 (0.60 to 1.22)
Total	55.11	0.14 (0.13 to 0.14)	1005.86 (747.96 to 1263.76)	202.90 (150.88 to 254.92)	18.25 (13.57 to 22.93)	3.68 (2.74 to 4.63)

The videos have been available for 7.1 and 10.6 months on average for adolescents and adults, respectively. Total gross impressions are based on the total number of intervals containing any alcohol content (233) and the number of intervals containing any tobacco content (47). Adolescents refer to the population aged between 11 and 18 years, while adults refer to the population aged 19 years or over.

## 

### Tobacco impressions

**Figure 1:**
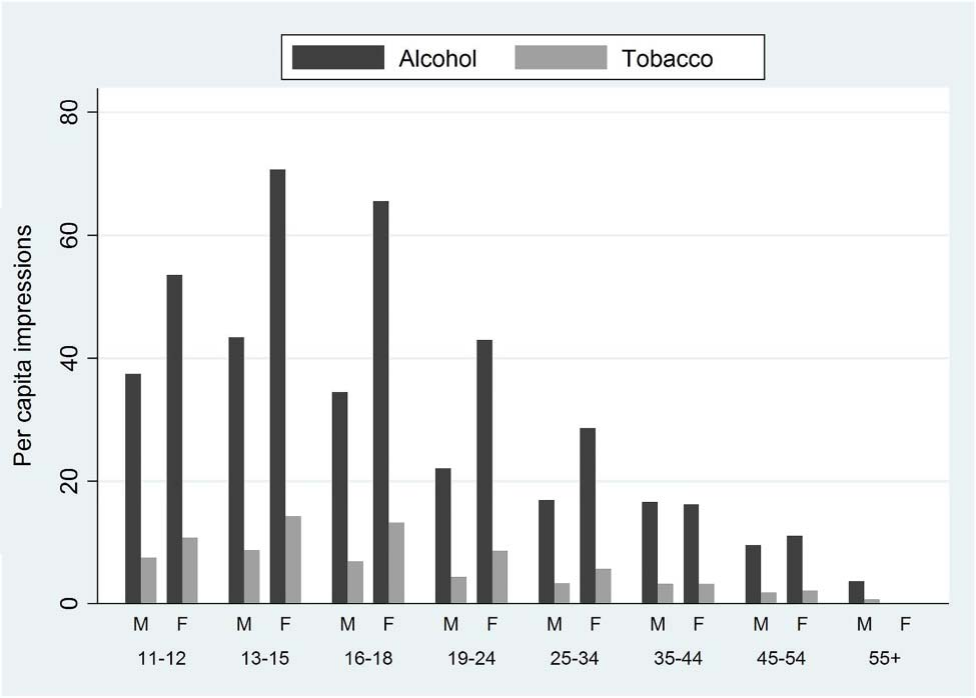
Per capita gross impressions for alcohol and tobacco content by gender and age group. M, male; F, female.

The highest number of gross tobacco impressions per capita occurred in the 13–18 age group, with each adolescent aged between 13–15 years and 16–18 years receiving an average of 11.48 (95% CI 9.83 to 13.14) and 10.50 (95% CI 8.44 to 12.56) impressions from these videos, respectively, while the figure per adult was 2.85 (95% CI 2.06 to 3.64) impressions per capita. Exposure in adolescents was around 65% higher in girls than in boys (13.04 (95% CI 10.71 to 15.38) and 7.86 (95% CI 6.52 to 9.19) impressions per capita, respectively (table 1), but the highest numbers of tobacco impressions per capita were delivered to girls aged 13–15 years (figure 1).

### Alcohol impressions

The pattern of exposure to alcohol impressions by age and gender was the same as for tobacco, but the number of impressions was approximately five times higher. Thus, an estimated average of 52.11 (95% CI 43.36 to 60.86) and 14.13 (95% CI 10.21 to 18.05) gross impressions of alcohol were delivered, respectively, to each adolescent and adult (table 1). In the age groups of 13–15 and 16–18 years, per capita impressions were, respectively, 56.93 (95% CI 48.73 to 65.13) and 52.07 (95% CI 41.85 to 62.29), and the highest exposure of 70.68 impressions per capita occurred in girls aged 13–15 years (figure 1).

### Tobacco and alcohol impressions delivered by individual music videos

Most of the music artists and bands (including main collaborators) were British (21), followed by American (11), then Dutch, Swedish, Canadian and from New Zealand (4, 3, 3 and 1, respectively). A breakdown of estimated adult and adolescent tobacco and alcohol impressions delivered by individual videos in the sample and music genre is provided in online supplementary table S2. For the whole population, the highest number of tobacco impressions was delivered by the video ‘Trumpets’ by Jason Derulo, which of a total of 36.80 (95% CI 32.31 to 41.30) million gross impressions delivered, 12.27 (95% CI 11.46 to 13.09) million were delivered to adolescents. However, per capita, this video delivered 2.07 (95% CI 1.94 to 2.21) tobacco impressions to adolescents compared with only 0.50 (95% CI 0.42 to 0.57) to the adult group. The same pattern was found in the videos ‘Love Me Again’ by John Newman and ‘Blurred Lines’ by Robin Thicke (featuring Pharrell Williams) which overall delivered the second and third most tobacco gross impressions; per capita, these videos both delivered more impressions to adolescents compared with the adult group at 0.75 (95% CI 0.70 to 0.81) vs 0.24 (95% CI 0.21 to 0.28) and 0.49 (95% CI 0.47 to 0.51) vs 0.25 (95% CI 0.24 to 0.27), respectively.

For the whole population, the highest number of alcohol gross impressions was generated by the video ‘Timber’ by Pitbull (featuring Ke$ha), delivering a total of 162.59 (95% CI 145.47 to 179.7) impressions including 44.47 (95% CI 41.71 to 47.22) million delivered to adolescents. However, per capita, the same video delivered 7.52 (95% CI 7.05 to 7.98) adolescent tobacco impressions compared with only 2.40 (95% CI 2.11 to 2.69) to the adult group. The videos ‘Best Song Ever’ by One Direction and ‘Drunk in Love’ by Beyoncé (featuring Jay-Z) again followed a similar pattern delivering the second and third most tobacco impressions to the whole population; per capita, these videos each delivered more adolescent alcohol impressions compared with the adult group at (7.99 (95% CI 7.59 to 8.40) vs 2.01 (95% CI 1.77 to 2.25) and 5.48 (95% CI 5.09 to 5.88) vs 1.99 (95% CI 1.74 to 2.25)), respectively. More tobacco and alcohol content, per capita, is therefore reaching adolescents than adults.

## Discussion

This is the first study to estimate the extent to which music videos deliver tobacco or alcohol imagery and lyrics to adults and adolescents. It demonstrates that this small sample of videos, which was among the most popular in the UK over a 12-week period from late 2013, delivered over 1.2 billion 10 s intervals containing tobacco or alcohol content to the British population. Approximately one-third of these impressions were delivered to people aged 11–18, and per capita rates in this age group were nearly four times higher than in adults. Impressions per capita were also much higher in girls than in boys. If these levels of exposure were typical then in 1 year, music videos would be expected to deliver over four billion impressions of alcohol, and nearly one billion of tobacco, in Britain alone. Further, the number of impressions has been calculated on the basis of on one viewing only; however, many of the videos had been watched multiple times (see online supplementary appendix 1), so this number is likely to be much bigger.

Our findings are consistent with previous research that found a larger sample of movies delivered 13.9 billion gross smoking impressions, an average of 665 to each US adolescent aged 10–14 years, and adds to the wider substance exposure research by including, for the first time, alcohol content reach.^19^ Since many of the popular songs in our sample were globally as well as nationally successful, it is evident that music videos are a major global as well as national medium of exposure to imagery that promotes smoking and alcohol consumption.

Legislation prohibiting paid-for placement of branded tobacco products has been in place since 2002 in the UK, and many other countries apply similar restrictions. It is therefore unlikely that tobacco brand appearances in UK-made music videos are funded by the tobacco industry, though the possibility remains that funding of generic imagery still occurs. The association between exposure to smoking imagery and smoking in films and uptake of smoking occurs even when branding is absent, which suggests that the effect of such exposure on the likelihood of smoking initiation is unrelated to branding.^21^ Tobacco companies still therefore have an incentive to encourage inclusion of smoking in films and music videos, but it seems likely that legal restraints on funding at least in part explain the much lower levels of tobacco than alcohol content.

Alcohol promotion in the UK is regulated only by ASA, the Portman Group and alcohol industry voluntary codes of practice, and as we have reported previously many alcohol companies have overt associations with artists who in turn feature their products in music videos.^9^ Such paid-for content directly contravenes several of these codes of practice such as associating alcohol with rites of passage into adulthood and drinking to excess.^15^ ^22^ ^23^ The lower levels of tobacco exposure we found indicate that similar controls on alcohol promotion could significantly reduce exposure of children through this medium, but would not completely remove it.

One relevant limitation of this research is that we have selected our sample of videos from the top UK music chart, which are not necessarily the most watched music videos on *YouTube*. However, we argue that a British audience is most likely to watch the videos associated with the most popular chart songs. Our survey of adults was carried out later than that of adolescents, which may have reduced the disparity between adult and adolescent exposure, because adults had more time in which to watch the videos. Conversely, we also acknowledge that the time differences could have influenced the adult results because of recall bias resulting in adult respondents forgetting if they had seen a video or not. Our 10 s interval monitoring is semiquantitative, and affords similar weight to any appearance within the 10 s interval, however long in duration. While there is currently no accepted means of coding the plethora of imagery that can occur in this medium, a more quantitative approach is an urgent developmental priority. Coverage bias is a problem for most survey methods including online. We used YouGov because its members’ panel has a sufficient coverage drawn from lower prevalence groups and because it uses purposive sampling.^24^ Further, the survey data were weighted on key demographics. Online surveys have overtaken telephone surveys with similar response rates to face-to-face interviewing and provide social distance which reduces respondent reluctance to reveal sensitive information (in this case adolescent smoking and drinking behaviour) and YouGov scans all responses for inattentiveness (eg, straight line ticking).^24^

Music videos are almost unregulated for their content. While films are age classified in most countries, and television content is subject to controls on what is broadcast during periods when children are likely to be watching, music videos are subject to none of these restraints. The British Board of Film Classification has consulted on introducing an age-rating system for music videos made in the UK, but this is a global problem and at present only includes drug misuse, dangerous behaviour presented as safe, bad language, sexual behaviour and nudity, and threatening behaviour and violence. Owing to the obvious health implications for adolescents, we suggest that overly positive portrayals of both alcohol and tobacco in music videos should be included in both the drug misuse and dangerous behaviour presented as safe rating categories. One means of reducing impact at the national level would be to require antismoking/alcohol messages to be broadcast immediately before any video containing smoking or alcohol, or to apply the practice on tobacco in Indian cinema of publishing health warnings in subtitles over the video. However, a more effective alternative may be to cut out this content at source, through negotiation with the video makers and publishers. Disney films has now announced that it will no longer make films including smoking.^25^ We need a similar degree of social responsibility across the tobacco, alcohol and music industries. It is well established that young people exposed to depictions of tobacco and alcohol content in films are more likely to start smoking or to consume alcohol;^1–8^ therefore, future research should focus on exploring the behavioural effects of exposure to similar content in music videos through large-scale longitudinal studies.

**What is already known on this subject**

- Adolescent exposure to alcohol and tobacco in the media are determinants of subsequent alcohol and tobacco use.
- The extent of potential exposure has been transformed over the past decade by the emergence of social media, in which exposure to protobacco content has also been linked to favourable attitudes towards tobacco, including intention to smoke, in young non-smokers.

**What this study adds**

- This is the first study to provide a British population estimate of adult and adolescent exposure to alcohol and tobacco content in digital music videos.
- Our findings provide new evidence that significant numbers of British adolescents are being exposed to both visual and lyrical tobacco and alcohol content in popular online digital music videos.
- Per capita exposure rates are much higher in adolescents than in adults and higher in girls than in boys.
- Alcohol and tobacco content in music videos is therefore a significant health hazard that requires appropriate regulatory control.

## Supplementary Material

Web appendix 1

Web table 1

Web table 2

## Footnotes

i We also provide information on gross impressions in millions per capita on music video watching ‘more than once’ by age and gender in online supplementary table S1b.
