# Supplementary material for: Adult and adolescent exposure to tobacco and alcohol content in contemporary YouTube music videos in Great Britain: a population estimate
Source: J Epidemiol Community Health. 2016 Jan 14;70(5):488–92. doi: 10.1136/jech-2015-206402 (PMC4853525; doi:10.1136/jech-2015-206402)
Supplement: Web appendix 1 [file jech-2015-206402-s1.pdf]

Appendix 1: General Characteristics of the Sample

	Percentage watched any (95% CI)	Percentage rewatched any (95% CI)	Average number of videos watched (95% CI)	Observations [%]
**Adolescents**	81.3 (79.7-83.0)	86.9 (85.3-88.5)	7.2 (6.8-7.5)	2,068 [100%]
Female	90.0 (88.2-91.8)	89.4 (87.4-91.4)	8.9 (8.4-9.3)	1,058 [48.8%]
Male	72.3 (69.5-75.0)	83.3 (81.0-86.4)	5.4 (5.0-5.7)	1,010 [51.2%]
**Adults**	40.4 (38.3-42.4)	75.5 (72.7-78.3)	1.9 (1.8-2.1)	2,232 [100%]
Female	43.1 (40.3-46.0)	73.9 (70.0-77.7)	2.3 (2.1-2.6)	1,180 [47.1%]
Male	37.7 (34.3-40.2)	77.6 (73.4-81.7)	1.5 (1.3-1.7)	1,052 [52.9%]
**Age Groups**				
11-12	78.0 (74.5-81.5)	88.3 (85.2-91.4)	6.2 (5.6-6.7)	537 [12.5%]
13-15	81.6 (79.0-84.3)	88.2 (85.2-90.7)	7.8 (7.3-8.4)	811 [18.9%]
16-18	83.5 (80.8-86.2)	84.5 (81.6-87.4)	7.2 (6.6-7.7)	720 [16.7%]
19-24	74.5 (69.4-79.6)	80.8 (75.4-86.1)	4.8 (4.1-5.4)	286 [6.7%]
25-34	62.5 (56.9-68.0)	77.0 (70.9-83.2)	3.3 (2.7-3.8)	293 [6.8%]
35-44	52.2 (47.1-57.3)	73.7 (67.5-80.0)	2.2 (1.8-2.7)	372 [8.7%]
45-54	35.4 (31.1-39.7)	73.7 (66.9-80.4)	1.4 (1.1-1.7)	472 [10.9%]
55+	17.8 (15.2-20.4)	70.1 (32.6-77.7)	0.6 (0.5-0.8)	809 [18.8%]
