# Supplementary material for: Adult and adolescent exposure to tobacco and alcohol content in contemporary YouTube music videos in Great Britain: a population estimate
Source: J Epidemiol Community Health. 2016 Jan 14;70(5):488–92. doi: 10.1136/jech-2015-206402 (PMC4853525; doi:10.1136/jech-2015-206402)
Supplement: Web table 1 [file jech-2015-206402-s2.pdf]

ONLINE SUPPLEMENTARY MATERIAL

			Gross Impressions (in millions)		Gross Impressions (per capita)	
	Population (in millions)	Proportion Viewed (95% CI)	Alcohol (95% CI)	Tobacco (95% CI)	Alcohol (95% CI)	Tobacco (95% CI)
**Adolescents**	5.91	0.136 (0.133-0.139)	187.26 (183.67-190.86)	37.77 (37.05-38.50)	31.66 (31.06-32.07)	6.39 (6.26-6.51)
Female	2.88	0.171 (0.167-0.175)	114.95 (112.26-117.64)	23.19 (22.64-23.73)	39.89 (38.95-40.82)	8.05 (7.86-8.23)
Male	3.03	0.099 (0.096-0.102)	69.89 (67.59-72.19)	14.10 (13.63-14.56)	23.05 (22.29-23.81)	4.65 (4.50-4.80)
**Adults**	49.20	0.034 (0.033-0.035)	391.28 (376.01-406.54)	78.93 (75.85-82.01)	7.95 (7.64-8.26)	1.60 (1.54-1.67)
Female	25.18	0.042 (0.040-0.044)	244.41 (232.58-256.23)	49.30 (46.92-51.69)	9.71 (9.24-10.18)	1.96 (1.86-2.05)
Male	24.02	0.026 (0.024-0.027)	143.79 (134.33-153.25)	29.00 (27.10-30.91)	5.99 (5.59-6.38)	1.21 (1.13-1.29)
**Age Groups**						
11-12	1.38	0.124 (0.119-0.129)	40.02 (38.44-41.61)	8.07 (7.75-8.39)	28.99 (27.84-30.14)	5.85 (5.62-6.08)
13-15	2.21	0.146 (0.142-0.150)	75.25 (73.04-77.47)	15.18 (14.73-15.63)	34.04 (33.03-35.04)	6.87 (6.66-7.07)
16-18	2.32	0.133 (0.129-0.137)	71.97 (69.59-74.34)	14.52 (14.04-15.00)	30.99 (29.96-32.01)	6.25 (6.04-6.46)
19-24	5.12	0.087 (0.087-0.081)	103.58 (96.70-110.47)	20.89 (19.51-22.28)	20.21 (18.87-21.56)	4.08 (3.81-4.35)
25-34	8.68	0.058 (0.054-0.063)	118.16 (108.56-127.76)	23.84 (21.90-25.77)	13.62 (12.51-14.72)	2.75 (2.52-2.97)
35-44	8.46	0.038 (0.035-0.041)	74.87 (68.10-81.65)	15.10 (13.74-16.47)	8.85 (8.05-9.65)	1.78 (1.62-1.95)
45-54	9.03	0.025 (0.023-0.028)	52.80 (47.55-58.05)	10.65 (9.59-11.71)	5.85 (5.27-6.43)	1.18 (1.06-1.30)
55+	17.90	0.010 (0.009-0.011)	42.70 (37.58-47.81)	8.61 (7.58-9.64)	2.39 (2.10-2.67)	0.48 (0.42-0.54)
**Total**	55.11	0.083 (0.082-0.085)	579.36 (539.57-619.16)	116.87 (108.87-124.89)	10.51 (9.79-11.23)	2.12 (1.97-2.27)

Note: The videos have been available for 7.1 and 10.6 months in average for adolescents and adults respectively. Total gross impressions are based on total number of intervals containing any alcohol content (233) and the number of intervals containing any tobacco content (47). Adolescents refer to the population between 11 and 18 years old, while adults to the population aged 19 years old or above.

Table 1b: Gross Impressions in Millions and Per Capita on Music Video Watching (more than once) for the UK population by Gender and Age Group.
