# Supplementary material for: Adult and adolescent exposure to tobacco and alcohol content in contemporary YouTube music videos in Great Britain: a population estimate
Source: J Epidemiol Community Health. 2016 Jan 14;70(5):488–92. doi: 10.1136/jech-2015-206402 (PMC4853525; doi:10.1136/jech-2015-206402)
Supplement: Web table 2 [file jech-2015-206402-s3.pdf]

| | Month since video was released | | Gross impression (in millions) | | | | Gross Impressions (per capita) | | | |
|---|---|---|---|---|---|---|---|---|---|---|
| | | | Adolescents | | Adults | | Adolescents | | Adults | |
| Genre/Music Video Title | Adoles-cents | Adults | Alcohol (95% CI) | Tobacco (95% CI) | Alcohol (95% CI) | Tobacco (95% CI) | Alcohol (95% CI) | Tobacco (95% CI) | Alcohol (95% CI) | Tobacco (95% CI) |
| Pop/R&B/Soul | 6.5 | 10.3 | 261.61 (257.11-266.11) | 38.25 (37.59-38.90) | 642.43 (620.58-664.28) | 93.92 (90.73-97.12) | 44.23 (43.47-44.99) | 6.47 (6.36-6.58) | 13.06 (12.61-13.50) | 1.91 (1.84-1.97) |
| The Story Of My Life (One Direction) | 5.0 | 8.5 | 7.14 (6.76-7.51) | 0 | 16.99 (15.04-18.95) | 0 | 1.21 (1.14-1.27) | 0 | 0.35 (0.31-0.39) | 0 |
| Royals (Lorde) | 10.8 | 14.3 | 16.38 (15.41-17.35) | 2.05 (1.93-2.17) | 37.21 (32.43-41.99) | 4.65 (4.05-5.25) | 2.77 (2.61-2.93) | 0.35 (0.33-0.37) | 0.76 (0.66-0.85) | 0.09 (0.08-0.11) |
| Love Me Again (John Newman) | 10.9 | 14.4 | 22.31 (20.65-23.97) | 4.46 (4.13-4.79) | 59.84 (51.48-68.21) | 11.97 (10.30-13.64) | 3.77 (3.49-4.05) | 0.75 (0.70-0.81) | 1.22 (1.05-1.39) | 0.24 (0.21-0.28) |
| Timber (Pitbull ft Kesha) | 4.2 | 7.7 | 44.47 (41.71-47.22) | 0 | 118.12 (103.76-132.49) | 0 | 7.52 (7.05-7.98) | 0 | 2.40 (2.11-2.69) | 0 |
| One More Sleep (Leona Lewis) | 4.1 | 7.6 | 5.67 (5.05-6.28) | 0 | 13.73 (10.93-16.53) | 0 | 0.96 (0.85-1.06) | 0 | 0.28 (0.22-0.34) | 0 |
| Can We Dance (The Vamps) | 7.9 | 11.4 | 9.61 (8.79-10.43) | 0 | 16.58 (13.29-19.86) | 0 | 1.63 (1.49-1.76) | 0 | 0.34 (0.27-0.40) | 0 |
| Best Song Ever (One Direction) | 8.4 | 11.9 | 47.27 (44.88-49.67) | 0 | 98.84 (86.90-110.77) | 0 | 7.99 (7.59-8.40) | 0 | 2.01 (1.77-2.25) | 0 |
| You're Nobody 'Til | 6.5 | 10.0 | 11.97 | 0 | 25.79 | 0 | 2.02 | 0 | 0.52 | 0 |

| | | | | | | | | | | |
|---|---|---|---|---|---|---|---|---|---|---|
| Somebody Loves You (James Arthur) | | | (11.01-12.93) | | (21.48-30.09) | | (1.86-2.19) | | (0.44-0.61) | |
| It's My Party (Jessie J) | 7.9 | 11.4 | 13.89 (13.05-14.74) | 0 | 25.15 (21.43-28.87) | 0 | 2.35 (2.21-2.49) | 0 | 0.51 (0.44-0.59) | 0 |
| Juliet (Lawson) | 7.1 | 10.6 | 18.12 (16.18-20.06) | 4.12 (3.68-4.56) | 27.16 (20.13-34.18) | 6.17 (4.57-7.77) | 3.06 (2.74-3.39) | 0.70 (0.62-0.77) | 0.55 (0.41-0.69) | 0.13 (0.09-0.16) |
| Trumpets (Jason Derulo) | 4.9 | 8.4 | 5.26 (4.91-5.61) | 12.27 (11.46-13.09) | 10.51 (8.94-12.09) | 24.53 (20.85-28.21) | 0.89 (0.83-0.95) | 2.07 (1.94-2.21) | 0.21 (0.18-0.25) | 0.50 (0.42-0.57) |
| Bonfire Heart (James Blunt) | 7.2 | 10.7 | 4.89 (4.42-5.36) | 0.98 (0.88-1.07) | 12.56 (10.32-14.81) | 2.51 (2.06-2.96) | 0.83 (0.75-0.91) | 0.17 (0.15-0.18) | 0.26 (0.21-0.30) | 0.05 (0.04-0.06) |
| Braveheart (Neon Jungle) | 3.7 | 7.2 | 0 | 0.75 (0.66-0.83) | 0 | 1.34 (1.01-1.68) | 0 | 0.13 (0.11-0.14) | 0 | 0.03 (0.02-0.03) |
| R U Crazy (Conor Maynard) | 7.3 | 10.8 | 2.40 (2.20-2.61) | 6.01 (5.49-6.52) | 3.04 (2.33-3.75) | 7.60 (5.84-9.37) | 0.41 (0.37-0.44) | 1.02 (0.93-1.10) | 0.06 (0.05-0.08) | 0.15 (0.12-0.19) |
| Work B**ch (Britney Spears) | 6.1 | 9.6 | 5.10 (4.56-5.63) | 0 | 16.13 (13.35-18.92) | 0 | 0.86 (0.77-0.95) | 0 | 0.33 (0.27-0.38) | 0 |
| Drunk In Love (Beyonce ft, Jay Z) | 3.5 | 7.0 | 32.43 (30.08-34.78) | 1.54 (1.43-1.66) | 98.13 (85.55-110.71) | 4.67 (4.07-5.27) | 5.48 (5.09-5.88) | 0.26 (0.24-0.28) | 1.99 (1.74-2.25) | 0.09 (0.08-0.11) |
| Hard Out Here (Lilly Allen) | 4.7 | 8.2 | 9.55 (8.67-10.43) | 0 | 22.22 (18.20-26.23) | 0 | 1.62 (1.47-1.76) | 0 | 0.45 (0.37-0.53) | 0 |
| Blurred Lines (Robin Thicke ft, Pharrell Williams) | 12.6 | 16.1 | 11.67 (11.16-12.18) | 2.92 (2.79-3.04) | 50.17 (46.61-53.73) | 12.54 (11.65-13.43) | 1.97 (1.89-2.06) | 0.49 (0.47-0.51) | 1.02 (0.95-1.09) | 0.25 (0.24-0.27) |

| | | | | | | | | | | |
|---|---|---|---|---|---|---|---|---|---|---|
| Dance/House | 5.9 | 9.5 | 30.35 (29.22-34.48) | 3.25 (3.13-3.37) | 65.21 (60.26-70.17) | 6.99 (6.46-7.52) | 5.13 (4.94-5.32) | 0.55 (0.53-0.57) | 1.33 (1.22-1.43) | 0.14 (0.13-0.15) |
| Under Control (Calvin Harris & Alesso ft, Hurts) | 5.4 | 8.9 | 2.13 (1.88-2.38) | 0 | 3.90 (2.92-4.88) | 0 | 0.36 (0.32-0.40) | 0 | 0.08 (0.06-0.10) | 0 |
| Animals (Martin Garrix) | 9.6 | 13.1 | 1.84 (1.65-2.02) | 0 | 3.39 (2.65-4.14) | 0 | 0.31 (0.28-0.34) | 0 | 0.07 (0.05-0.08) | 0 |
| Rather Be (Clean Bandit ft, Jess Glynne) | 3.9 | 7.4 | 3.10 (2.81-3.39) | 1.03 (0.94-1.13) | 8.00 (6.61-9.39) | 2.67 (2.20-3.13) | 0.52 (0.47-0.57) | 0.17 (0.16-0.19) | 0.16 (0.13-0.19) | 0.05 (0.04-0.06) |
| Hey Brother (Avicii) | 3.7 | 7.3 | 2.07 (1.95-2.20) | 4.15 (3.90-4.39) | 5.03 (4.41-5.64) | 10.05 (8.81-11.29) | 0.35 (0.33-0.37) | 0.70 (0.66-0.74) | 0.10 (0.09-0.11) | 0.20 (0.18-0.23) |
| You Make Me (Avicii) | 6.6 | 10.1 | 22.70 (20.89-24.52) | 0 | 47.96 (39.89-56.04) | 0 | 3.84 (3.53-4.15) | 0 | 0.97 (0.81-1.14) | 0 |
| Boo Yah (Showtek/ We Are Loved/ Wilson) | 6.5 | 10.0 | 0.87 (0.74-1.00) | 0 | 0.93 (0.53-1.32) | 0 | 0.15 (0.12-0.17) | 0 | 0.02 (0.02-0.01) | 0 |
| Electronic/Alternative | 8.4 | 11.9 | 17.24 (16.45-18.03) | 15.08 (14.40-15.77) | 33.50 (30.21-36.80) | 29.32 (26.43-32.20) | 2.91 (2.78-3.05) | 2.55 (2.43-2.67) | 0.68 (0.61-0.75) | 0.60 (0.54-0.65) |
| Of The Night (Bastille) | 5.8 | 9.3 | 3.56 (3.25-3.87) | 4.75 (4.34-5.16) | 5.42 (4.27-6.57) | 7.23 (5.69-8.77) | 0.60 (0.55-0.65) | 0.80 (0.73-0.87) | 0.11 (0.09-0.13) | 0.15 (0.12-0.18) |
| Pompeii (Bastille) | 14.5 | 18.0 | 7.72 (7.24-8.20) | 1.93 (1.81-2.05) | 18.16 (15.80-20.53) | 4.54 (3.95-5.13) | 1.31 (1.22-1.39) | 0.33 (0.31-0.35) | 0.37 (0.32-0.42) | 0.09 (0.08-0.10) |
| Count On Me (Chase & Status) | 6.4 | 9.9 | 2.23 (1.93-2.53) | 4.46 (3.86-5.06) | 3.88 (2.74-5.02) | 7.76 (5.49-10.03) | 0.38 (0.33-0.43) | 0.75 (0.65-0.86) | 0.08 (0.06-0.10) | 0.16 (0.11-0.20) |

| | | | | | | | | | | |
|---|---|---|---|---|---|---|---|---|---|---|
| Afterglow (Wilkinson) | 6.8 | 10.3 | 3.17 (2.78-3.57) | 0.64 (0.56-0.71) | 5.29 (3.81-6.77) | 1.06 (0.76-1.35) | 0.54 (0.47-0.60) | 0.11 (0.09-0.12) | 0.11 (0.08-0.14) | 0.02 (0.02-0.03) |
| Hip-hop | 8.9 | 12.4 | 17.89 (17.03-18.75) | 4.97 (4.73-5.21) | 24.70 (21.67-27.72) | 6.86 (6.02-7.70) | 3.02 (2.88-3.17) | 0.84 (0.80-0.88) | 0.50 (0.44-0.56) | 0.14 (0.12-0.16) |
| Berzerk (Eminem) | 6.8 | 10.3 | 0 | 1.99 (1.80-2.18) | 0 | 2.34 (1.71-2.96) | 0 | 0.34 (0.30-0.37) | 0 | 0.05 (0.03-0.06) |
| Hold On  We're Going Home (Drake ft, Majid Jordan) | 6.3 | 9.8 | 13.85 (12.52-15.19) | 2.97 (2.68-3.25) | 17.59 (13.08-22.10) | 3.77 (2.80-4.74) | 2.34 (2.12-2.57) | 0.50 (0.45-0.55) | 0.36 (0.27-0.45) | 0.08 (0.06-0.10) |
| Rap God (Eminem) | 4.2 | 7.7 | 0.92 (0.83-1.02) | 0 | 1.21 (0.90-1.53) | 0 | 0.16 (0.14-0.17) | 0 | 0.02 (0.02-0.03) | 0 |
| Same Love (Macklemore & Ryan Lewis ft, Mary Lambert) | 18.2 | 21.7 | 3.20 (2.91-3.49) | 0 | 5.55 (4.39-6.72) | 0 | 0.54 (0.49-0.59) | 0 | 0.11 (0.09-0.14) | 0 |

**Note:** Population of adolescents is 5.91 million and adults, 49.20 million.

**Online Table 2:** Gross Impressions in Millions and Per Capita for Adults and Adolescents by Music Video and Genre
